# Precise pancreatic necrosectomy by step-up approach

**DOI:** 10.17179/excli2021-4131

**Published:** 2021-08-16

**Authors:** Sudharsanan Sundaramurthi, Amudhan Kannan, Rajkumar Nagarajan, Shanmugam Dasarathan, Kadambari Dharanipragada

**Affiliations:** 1 Department of Surgery, Jawaharlal Institute of Postgraduate Medical Education and Research (JIPMER), Puducherry, India; 2 Medical student, Jawaharlal Institute of Postgraduate Medical Education and Research (JIPMER), Puducherry, India

## ⁯⁯⁯⁯

***Dear Editor,***

Necrotizing pancreatitis is a concerning clinical condition diagnosed with the help of contrast-enhanced computed tomography in which the necrosed part will not show enhancement. It constitutes about 10 %-20 % of patients who present with acute pancreatitis and carries high morbidity and mortality. One of the most important factors associated with mortality is the infection of the necrotic tissue, which almost doubles the mortality rate from 10-15 % to around 30-40 % in patients with necrotizing pancreatitis (Petrov et al., 2010[4]; Bugiantella et al., 2016[2]). The initial treatment of necrotizing pancreatitis includes fluid resuscitation, nutrition, and intensive care support. The timing of the debridement of the necrotic tissues plays a crucial role in the outcome of patients with necrotizing pancreatitis (Aranda-Narváez et al., 2014[1]). The best results in pancreatic necrosectomy are obtained by the “step-up” approach, where the patient is initially managed with conservative measures such as percutaneous drainage of pus and IV antibiotics. The surgery, if necessary, is delayed up to one month after the onset of the clinical symptoms. This method provides better demarcation of necrosis (conversion to “walled-off” pancreatic necrosis), involves less bleeding and less removal of surrounding viable tissues (van Baal et al., 2011[6]). 

**Figure 1:**
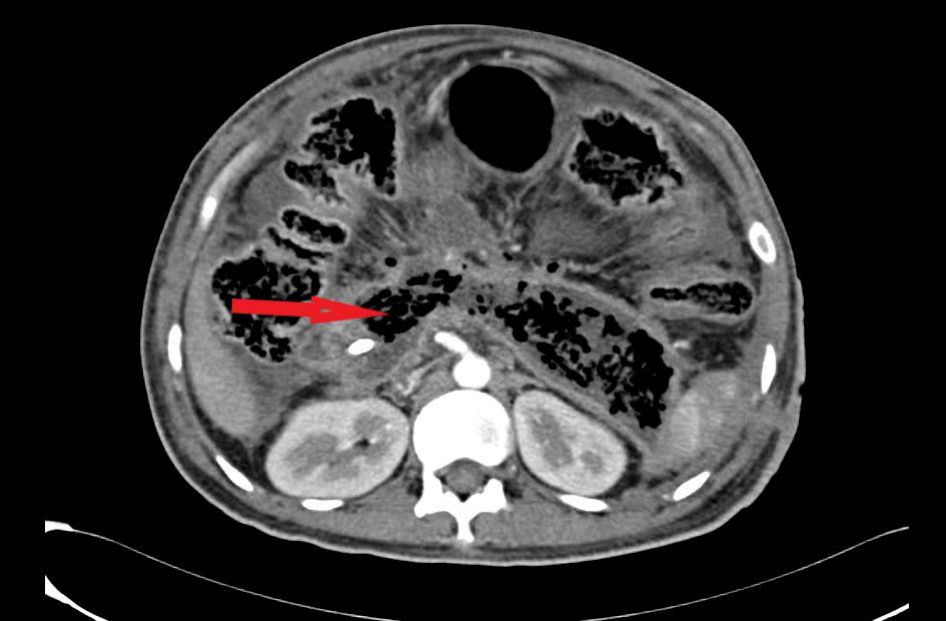
Contrast-enhanced computed tomography (CECT) image showing pancreatic necrosis. The red arrow shows less enhancement suggestive of necrosis.

**Figure 2:**
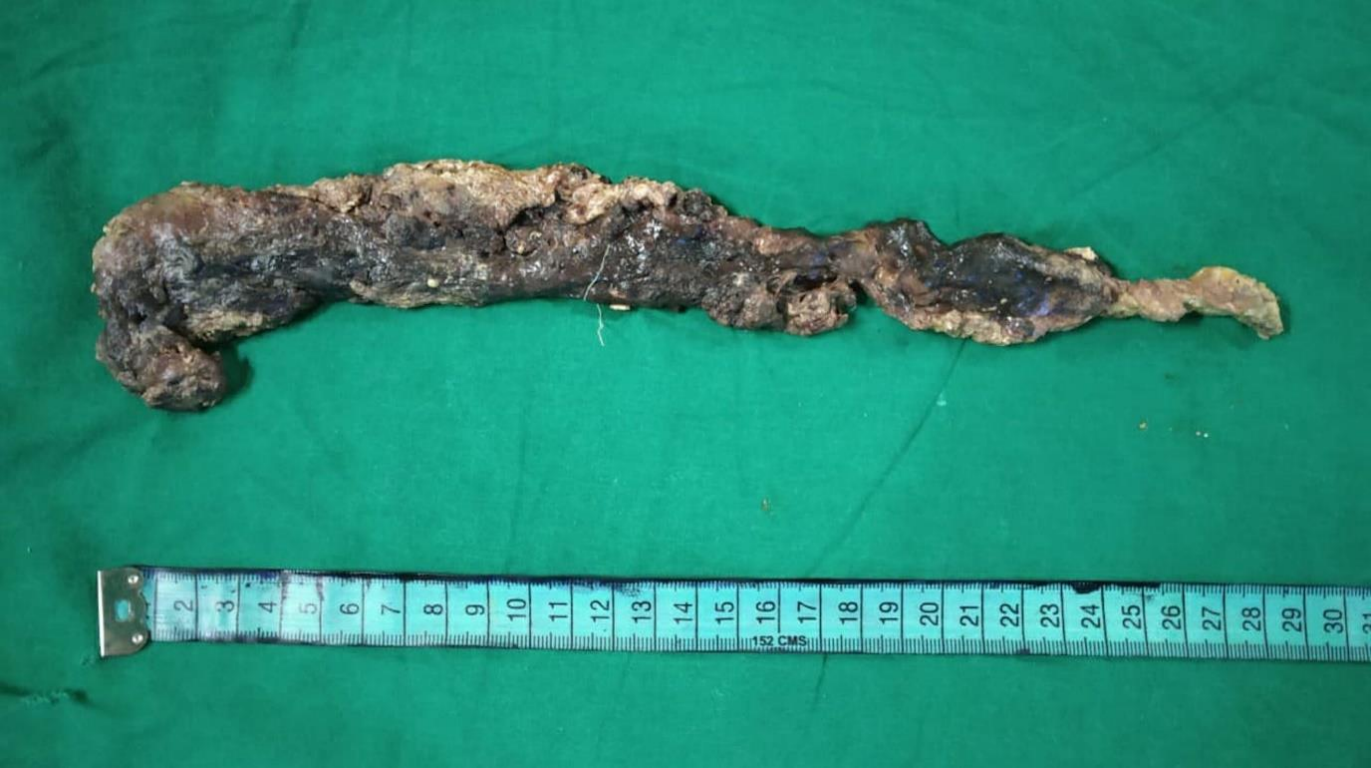
Pancreatic necrosectomy specimen

Recently, we read with great interest a systematic review and network meta-analysis by Ricci et al. (2021[5]) on the delayed treatment of infected necrotizing pancreatitis. We congratulate all the authors for their outstanding effort put into bringing this study to the literature. A total of seven studies were included. This meta-analysis evaluated the mortality rate in all the delayed surgical procedures like delayed debridement, the step-up approach using minimally invasive debridement, and the step-up approach using endoscopic debridement. The authors reported that the step-up approach using endoscopic debridement was associated with lower morbidity and mortality rates than other delayed procedures. The authors recommended using the step-up approach with endoscopic debridement for all patients with infected necrotizing pancreatitis (Ricci et al., 2021[5]). In our institute, we recently came across a 36-year-old alcoholic gentleman admitted to the emergency department with abdominal pain and vomiting for a one-week duration. Blood pressure was 100/60 mmHg. He had severe epigastric tenderness, upper abdominal guarding and tachycardia, blood pressure of 100/60 mmHg, and serum amylase of 1280 IU/L. Contrast-enhanced computed tomography (CECT) revealed more than 80 % necrosis of the pancreas associated with pus collections at the lesser sac and the pancreatic tail region. The CECT image is shown in Figure 1(Fig. 1). We followed the step-up approach perfectly without any deviation. The pus collection was drained using a pigtail catheter, and the patient was started on intravenous antibiotics based on the culture sensitivity pattern. There was persistent pus discharge in the range of 200 ml for more than a month and reduced tolerance to enteral feeds that prompted us to take the patient for open pancreatic necrosectomy. Intra-operatively, the entire pancreas, except for a thin rim all around, was found to be necrotic. The necrosectomy was done perfectly without any loss or lateral damage to the surrounding tissue. The necrotic tissues involving the pancreatic head, body, and tail were removed in toto. The completely removed specimen of the necrotic pancreas is shown in Figure 2(Fig. 2). Large bore drains were placed in the pancreatic area for lavage in the post-operative period. The patient made an uneventful recovery. 

In the meta-analysis mentioned above, the authors suggested that if the endoscopic approach is not available, a step-up approach with minimally invasive surgical debridement could be considered. Though open necrosectomy is rarely performed today, endoscopic or minimally invasive procedures are not available in our institute and the majority of the hospitals in India. The open necrosectomy was delayed for one month in this patient, which should have led to a walling-off of the pancreatic necrosis. This had led us to seamless dissection and ostomy creation between the necrosed walled-off part and other parts that ultimately resulted in complete and perfect removal of the entire necrotic pancreas without any lateral damage.

The classical immediate surgical approach or the “step-down” approach consists of early surgery when there is an established indication and, later on, more conservative treatment for the residual disease. The initial period of necrotizing pancreatitis is associated with a severe inflammatory response. Surgery during this phase will aggravate the multi-organ dysfunction and result in an increased rate of complications, such as bleeding and pancreatic fistula formation (da Costa et al., 2014[3]). Studies in the literature had reported a complete success rate of 55 % in the step-up approach when the initial management of infected pancreatic necrosis was done with percutaneous drainage. Additional surgery can also be avoided (van Baal et al., 2011[6]). Delaying surgical treatment by early percutaneous drainage can facilitate the use of minimally invasive techniques like endoscopy or laparoscopy for debridement of necrotic tissues. The mainstay of treatment of necrotizing pancreatitis should be a staged, multidisciplinary, step-up approach with minimally invasive procedures opted for pancreatic necrosectomy.
